# Prevalence and Clinical Implications of Scleroderma-Specific Autoantibodies in Seronegative Patients with Sicca Complaints

**DOI:** 10.31138/mjr.20230808.pa

**Published:** 2023-08-08

**Authors:** Nikolaos Marketos, Clio P. Mavragani

**Affiliations:** 1 Department of Physiology, Medical School of Athens, National and Kapodistrian University of Athens, Athens, Greece,; 2 Department of Pathophysiology, Medical School of Athens, National and Kapodistrian University of Athens, Athens, Greece

**Keywords:** Sjögren’s syndrome, sicca, Systemic sclerosis, autoantibodies

## Abstract

**Introduction:**

Previous studies have revealed the presence of anticentromere antibodies in patients with Sjögren’s syndrome (SS), predominantly in those serologically negative for antibodies against Ro/SSA and La/SSB antigens (seronegative). The prevalence and clinical significance of specific autoantibodies for Systemic Sclerosis (SSc) in seronegative patients with sicca complaints (dry eyes, dry mouth) have not yet been studied.

**Aim of the study:**

Investigate the prevalence and clinical significance of SSc-specific autoantibodies in seronegative patients with sicca complaints.

**Methods:**

Among 212 patients with sicca symptoms that were investigated at the Laboratory of Physiology, Medical School of Athens, National and Kapodistrian University of Athens between the years 2017–2020 for the presence of antibodies against Ro/SSA and La/SSB antigens, we found 106 patients that did not meet the criteria for SS following a thorough investigation. We have already performed serological tests of 13 specific autoantibodies for Scleroderma by using the test EUROLINE Systemic Sclerosis (Nucleoli) profile (IgG) (EUROIMMUNE Medizinische Labordiagnostika AG) in 51 of these patients. Additionally, their demographic, clinical, and laboratory data have been recorded.

**Results:**

The presence of the specific SSc-specific autoantibodies was noticed in 29/51 patients (57%). More precisely, in 7/29 patients (24%) we recorded NOR90 as well as Th/To, in 5/29 (17%) Ku & Ro52, in 4/29 (14%) CENP-B, in 3/29 (10%) CENP-A & RP155, in 2/29 (7%) RP11 & PM/Scl 75 and finally in 1/29 (3.5%) Scl70, PM/Scl 100 & Fibrillarin respectively. The subsequent follow-up of these patients revealed signs of pulmonary hypertension and interstitial lung disease in some cases.

**Conclusions:**

The high prevalence of Scleroderma-specific autoantibodies in seronegative sicca patients discloses those needing tighter follow-up and systematic treatment.

## BACKGROUND

Sjögren’s syndrome (SS) is a chronic autoimmune entity characterized by the infiltration of salivary and lacrimal glands by inflammatory cells and affects mainly middle-aged women leading to oral and ocular dryness.^1,2^ Various features have been observed in patients suffering from dry mouth and dry eyes, ranging from non-specific symptoms and signs such as fatigue and Raynaud’s phenomenon (RP), periepithelial infiltration leading to interstitial nephritis and bronchiolitis, and finally immune-complex mediated disease, such as peripheral neuropathy and glomerulonephritis. Except for cases needing immunomodulatory treatment, this disease runs an indolent course. Nevertheless, in a subgroup of patients with specific risk factors,^3^ the incidence of lymphoma is higher, sometimes requiring chemotherapy.^4,5^

SS can exist in the presence of other autoimmune diseases, such as Systemic Sclerosis (SSc).^6,7^ However, in recent years the attention of the scientific community is drawn especially to a subgroup of patients with SS and anticentromere antibodies (ACA[+]SS).^8^ This condition is considered by many as an overlap syndrome between SS and SSc. There are three areas of particular interest regarding these patients’ immunological and clinical profile: features that co-exist or differentiate them from SS and SSc patients, the risk of lymphoma, and possible prognostic factors of heavier disease burden.

The presence of ACA seems to confer in scleroderma features. It has been demonstrated that neither CENP-B nor CENP-C autoantibodies co-exist in SS patients in contrast to SSc(9). The different groups of ACA(+) SS, ACA(−) SS, and a subgroup of ACA(+) SS-limited cutaneous (lc)SSc were compared in a study by Salliot et al.^10^ The overlap group presented a milder scleroderma phenotype similar to ACA(−) SS regarding minor salivary gland biopsies (MSGB) and ACA(+) SS regarding serology. These results were confirmed by another study.^7^ Furthermore, similar findings were reported by Bournia et al. who investigated four subgroups: ACA(+) SS, ACA(−) SS, ACA(+) SSc/sicca(+), and ACA(+) SSc/sicca(−).^11^ The finding of ACA in SS was consistent with a lower prevalence of anti-Ro/SSA and anti-La/SSB antibodies than its ACA(−) counterparts and milder CREST (Calcinosis, RP, Esophageal Dysmotility, Sclerodactyly, Telangiectasias) phenotype compared to lcSSc. Similarly, Baer et al. showed worse exocrine glandular function, and higher RP incidence but a lower prevalence of anti-Ro, anti-La antibodies in SS ACA(+) patients.^12^ This intermediate clinical phenotype between SS and SSc was described in more detail in a retrospective study by Bournia et al.^13^

As expected, this overlapping group was not reported to run a higher lymphoma risk by these investigators.^11^ Controversially, Baldini et al.^14^ described a similar group of patients to have a higher prevalence of non-Hodgkin lymphoma. A more recent study by Notarstefano et al. confirmed that ACA positivity granted more often CREST features and an increased need for immunomodulatory drugs, but not a higher risk for MALT lymphoma.^15^ These data show that ACA(+) SS may exhibit more often features of SSc but seem to have a lower risk for lymphoma than ACA(−) SS.

Interestingly, it has also been postulated that as much as 25% of ACA(+) SS patients may eventually be classified as lcSSc on follow-up, further strengthening the connection between these two entities.^16^

Given these exciting results using ACA as a differentiating and comparison factor between SS and SSc patients, more profound insight into other specific SSc autoanti-bodies in SS patients becomes prominent. For example, patients with sicca symptoms (dry eyes, dry mouth) lacking antibodies against Ro/SSA and La/SSB antigens are considered seronegative. Although ACA are more often found in seronegative SS patients,^17^ the prevalence and clinical significance of the above-mentioned specific SSc autoantibodies in seronegative sicca patients has not yet been elucidated.

## SIGNIFICANCE

Specific autoantibodies in SSc characterise different clinical profiles, often helping prognosis and therapeutic decisions in these patients.

- Anti-TOPO/anti-Scl-70 signifies an increased risk for fibrosis, especially in the lungs,^18^ scleroderma renal crisis, and skin ulceration.^19^ Conversely, their absence indicates a better prognosis and survival.^20^
- ACA in lcSSc patients^21^ indicates a higher risk of pulmonary hypertension and gastrointestinal involvement.
- Anti- RNAP III or RP11/RP155 are evident in rapid skin fibrosis and renal crisis^22^ and are a risk factor for GAVE (gastric antral vascular ectasia).^23^ In addition, these patients seem to have been more often cancer diagnosed at the time of antibody positivity.^24^
- Anti-fibrillarin or anti-U3 RNP predisposes to multi-organ involvement. Pulmonary hypertension is more rapidly evolving than fibrosis,^25^ gastrointestinal tract involvement, myopathy, and peripheral neuropathy.^26^
- Anti-NOR90 in lcSSc indicates minor internal organ involvement,^27^ although it often is related to ILD.^28^
- Anti-Th/To (7-2 RNP, 8-2 RNP) are the most prevalent specific SSc autoantibodies in ANA (−) patients.^29^ They are associated with pulmonary fibrosis and hypertension, as well.^30^ These patients have the worst prognosis among lcSSc.^25^
- Anti-PM/Scl characterizes patients with overlap syndrome as well as myositis and arthritis.^31^ Subcutaneous calcification is encountered more often, but pulmonary fibrosis is seldom identified in those patients.^32^
- Anti-Ku (p70/p80) is often present in SLE patients, myositis, and arthritis.^33^ In addition, trigeminal neuralgia and thyroiditis are usually found in this group of patients,^34^ and they also have a higher cancer incidence.
- Anti-platelet-derived growth factor (PDGF) stimulating antibodies^35^ may have a role in the precipitation of fibrosis.^36^
- Finally, antibodies to Ro/SSA are present in diverse autoimmune diseases,^37^ but their role remains obscure.

In light of the association of the above mentioned antibodies to clinical phenotype of SSc, we aim to assess these antibodies in SS patients from a prognostic and therapeutic perspective.

## OBJECTIVES

Our group examines the prevalence and clinical significance of specific SSc autoantibodies in SS patients.

## STUDY PARTICIPANTS

The study will use serum from patients both in a retrospective and prospective manner. We examine the serum of patients already stored in the laboratory of the Department of Physiology, Medical School, National and Kapodistrian University of Athens, and new patients. Patients characterized as SS are all fulfilling the 2016 EULAR/ACR classification criteria for SS.^38^ An approximate total of 90 SS patients with lymphoma and 90 without will be compared. A subgroup of patients presenting with sicca complaints between January 2017 and December 2020 will be investigated in parallel. A subset of these patients will eventually be diagnosed as SS, another one having other autoimmune diagnoses, while a third one will be classified as undifferentiated. All patients must have signed informed consent before any investigation is performed.

## STUDY PROTOCOL

As mentioned above, we will test these patients’ serum for 13 specific SSc autoantibodies using the commercially available kit EUROLINE Systemic Sclerosis (Nucleoli) profile (IgG) EUROIMMUNE Medizinische Labordiagnostika AG. Blood samples from around 220 SS patients are stored in a refrigerator at −80°C in the lab of the Department of Physiology, Medical School of Athens, National and Kapodistrian University of Athens. The investigators will gather all necessary data regarding medical history, clinical assessment, laboratory findings, and especially details about fulfilling the 2016 EULAR/ACR classification criteria and immunological tests and treatment both in the past and present. Additionally, we will gather all demographic and clinical/laboratory data of these patients.

Once we gather the data, we will proceed with the evaluation, thus identifying a smaller group of patients according to their immunological profile. We will use the SPSS statistical program for the statistical analysis. To compare some laboratory parameters to typical values, we will use a t-test, while for group comparison, we will apply a paired t-test. Pearson’s correlation coefficient will be administered to evaluate SSc autoantibodies’ presence concerning clinical data and videocapillaroscopy results in case of a normal distribution; alternatively, Wilcoxon test if that is not the case. We will use ANOVA for the between-group analysis.

## PROGRESS

In the second year of the study, we have narrowed down comparing patient groups regarding specific SSc autoantibodies to the most significant SS lymphoma subgroup and their age- and sex-matched counterparts. Most importantly, we have preliminary results from the group presenting with sicca symptoms. We have identified 106 sicca patients at this time point, 51 of which SSc-specific autoantibody testing is performed. This group of patients is almost always serologically (anti-Ro, anti-La) and often histologically (Focus score<1) negative, misleading physicians to disregard their profound sicca complaints. We have so far identified a substantial number of these patients who show positivity in specific SSc autoantibodies. More specifically, the presence of SSc-specific autoantibodies was noticed in 29/51 patients (57%). The distribution of SSc-specific auto-antibodies in our patients is as follows: NOR90 in 7/29 (24%) patients, Th/To in 7/29 (24%), Ku in 5/29 (17%), Ro52 in 5/29 (17%), CENP-B in 4/29 (14%), CENP-A in 3/29 (10%), RP155 in 3/29 (10%), RP11 in 2/29 (7%), PM/Scl 75 in 2/29 (7%), and finally Scl70 in 1/29 (3.5%), PM/Scl 100 in 1/29 (3.5%), and Fibrillarin in 1/29 (3.5%), while 4/29 (14%) patients had positive anti-Ro/SSA antibodies, still without fulfilling 2016 SS classification criteria. Additionally, all salivary gland biopsies performed were negative, while in 9/29 patients (31%), radiological features of interstitial lung disease (ILD) were present. Further investigation of these patients with tests such as echocardiography, spirometry, and high-resolution chest computed tomography (HRCT) has led to findings that require treatment for pulmonary hypertension, among other conditions. One of these patients was eventually diagnosed with idiopathic pulmonary fibrosis (IPAF) and received proper therapy.

The etiopathogenic mechanisms explaining the sicca phenotype remain elusive. It has been postulated that the antibodies against the muscarinic acetylcholine receptor may be implicated in SS.^39^ Simultaneously, fibrosis and inflammation of the vasculature as a pathogenetic procedure may play a crucial role in sicca regarding SSc patients.^39^ Still, little is known about the pathogenesis of sicca in patients with ACA and other SSc-specific autoantibodies. Furthermore, the unmet need for prompt diagnosis and treatment of autoimmune diseases is increasingly recognised,^40^ urging for better diagnostic and prognostic tools.

## CONCLUSION

Our cohort’s preliminary results show a high prevalence of Scleroderma-specific autoantibodies in patients under investigation for sicca complaints. This information will prove helpful in clinical practice by revealing those sicca patients who lack substantial evidence of clear-cut rheumatological diseases but have signs of organ dysfunction needing specific treatment, changing their differential diagnosis practice and follow-up routine.

## References

[1] Mavragani CP Moutsopoulos HM. Sjögren syndrome Review. CMAJ 2014;186(15):579–86. 10.1503/cmaj.122037 PMC4203623 24566651

[2] Skopouli FN Dafni U Ioannidis JPA Moutsopoulos HM. Clinical evolution, and morbidity and mortality of primary Sjogren’s syndrome. Semin Arthritis Rheum 2000;29(5):296–304. 10805354 10.1016/s0049-0172(00)80016-5

[3] Fragkioudaki S Mavragani CP Moutsopoulos HM. Predicting the risk for lymphoma development in Sjogren syndrome: An easy tool for clinical use. Medicine 2016;95(25):e3766. 27336863 10.1097/MD.0000000000003766 PMC4998301

[4] Voulgarelis M Giannouli S Anagnostou D Tzioufas AG. Combined therapy with rituximab plus cyclophosphamide/doxorubicin/vincristine/prednisone (CHOP) for Sjögren’s syndrome-associated B-cell aggressive non-Hodgkin’s lymphomas. Rheumatology 2004;43(8):1050–3. 15187246 10.1093/rheumatology/keh248

[5] Voulgarelis M Giannouli S Tzioufas AG Moutsopoulos HM. Long term remission of Sjögren’s syndrome associated aggressive B cell non-Hodgkin’s lymphomas following combined B cell depletion therapy and CHOP (cyclophosphamide, doxorubicin, vincristine, prednisone). Ann Rheum Dis 2006;65(8):1033–7. 16322082 10.1136/ard.2005.046193 PMC1798235

[6] Lawley TJ Gary L Moutsopoulos HM. Scleroderma, Sjogren-like Syndrome, and Chronic GGraft-Versus-HostDisease. Ann Intern Med 1977;87:707–9. 22307 10.7326/0003-4819-87-6-707

[7] Salliot C Mouthon L Ardizzone M Sibilia J Guillevin L Mariette X. Sjögren’s syndrome is associated with and not secondary to systemic sclerosis. Rheumatology 2007;46(2):321–6. 16877466 10.1093/rheumatology/kel252

[8] Katano K Kawano M Koni I Sugai S Muro Y. Clinical and laboratory features of anticentromere antibody positive primary Sjögren’s syndrome. J Rheumatol 2001;28(10):2238–44. 11669163

[9] Gelber AC Pillemer SR Baum BJ Wigley FM Hummers LK Morris S Distinct recognition of antibodies to centromere proteins in primary Sjögren’s syndrome compared with limited scleroderma. Ann Rheum Dis 2006;65(8):1028–32. 16414973 10.1136/ard.2005.046003 PMC1798261

[10] Salliot C Gottenberg JE Bengoufa D Desmoulins F Miceli-Richard C Mariette X. Anticentromere antibodies identify patients with Sjögren’s syndrome and autoimmune overlap syndrome. J Rheumatol 2007;34(11):2253–8. 17937465

[11] Bournia VKK Diamanti KD Vlachoyiannopoulos PG Moutsopoulos HM. Anticentromere antibody positive Sjögren’s Syndrome: A retrospective descriptive analysis. Arthritis Res Ther 2010;12(2). 10.1186/ar2958 PMC2888196 20302639

[12] Baer AN Medrano L McAdams-DeMarco M Gniadek TJ. Association of Anticentromere Antibodies With More Severe Exocrine Glandular Dysfunction in Sjögren’s Syndrome: Analysis of the Sjögren’s International Collaborative Clinical Alliance Cohort. Arthritis Care Res 2016;68(10):1554–9. 10.1002/acr.22859 PMC5429972 26867144

[13] Bournia VK Vlachoyiannopoulos PG. Subgroups of Sjögren syndrome patients according to serological profiles. J Autoimmun 2012;39(1–2):15–26. 22575069 10.1016/j.jaut.2012.03.001

[14] Baldini C Mosca M Della Rossa A Pepe P Notarstefano C Ferro F Overlap of aca-positive systemic sclerosis and Sjögren’s syndrome: A distinct clinical entity with mild organ involvement but at high risk of lymphoma. Clin Exp Rheumatol 2013;31(2):0273–80. 23343785

[15] Notarstefano C Croia C Pontarini E Lucchesi D Sutcliffe N Tappuni A A clinical and histopathological analysis of the anti-centromere antibody positive subset of primary Sjögren’s syndrome. Clin Exp Rheumatol 2018;36:S145–9. 30156540

[16] Ramos-Casals M Nardi N Brito-Zerón P Aguiló S Gil V Delgado G Atypical Autoantibodies in Patients with Primary Sjögren Syndrome: Clinical Characteristics and Follow-Up of 82 Cases. Semin Arthritis Rheum 2006;35(5):312–21. 16616154 10.1016/j.semarthrit.2005.12.004

[17] Park Y Lee J Koh JH Choe JY Sung YK Lee SS Clinical influences of anticentromere antibody on primary Sjögren’s syndrome in a prospective Korean cohort. Korean J Intern Med 2021 Nov;36(6):1492–1503. 32829574 10.3904/kjim.2020.146 PMC8588972

[18] Ho KT Reveille JD. The clinical relevance of autoantibodies in scleroderma. Arthritis Res Ther 2003;5(2):80–93. 12718748 10.1186/ar628 PMC165038

[19] Bohelay G Blaise S Levy P Claeys A Baudot N Cuny JF Lower-limb ulcers in systemic sclerosis: A multicentre retrospective case-control study. Acta Dermato-Venereologica 2018;98(7):677–82. 29648670 10.2340/00015555-2939

[20] Wirz EG Jaeger VK Allanore Y Riemekasten G Hachulla E Distler O Incidence and predictors of cutaneous manifestations during the early course of systemic sclerosis: A 10-year longitudinal study from the EUSTAR database. Ann Rheum Dis 2016;75(7):1285–92. 26232495 10.1136/annrheumdis-2015-207271

[21] Steen VD Powell DL Medsger TA. Clinical correlations and prognosis based on serum autoantibodies in patients with systemic sclerosis. Arthritis Rheum 1988;31(2):196–203. 3348823 10.1002/art.1780310207

[22] Hamaguchi Y Kodera M Matsushita T Hasegawa M Inaba Y Usuda T Clinical and immunologic predictors of scleroderma renal crisis in Japanese systemic sclerosis patients with anti-RNA polymerase III autoantibodies. Arthritis Rheumatol 2015;67(4):1045–52. 25512203 10.1002/art.38994

[23] Ghrénassia E Avouac J Khanna D Derk CT Distler O Suliman YA Prevalence, correlates and outcomes of gastric antral vascular ectasia in systemic sclerosis: A EUSTAR case-control study. J Rheumatol 2014;41(1):99–105. 24293584 10.3899/jrheum.130386

[24] Lazzaroni MG Cavazzana I Colombo E Dobrota R Hernandez J Hesselstrand R Malignancies in patients with anti-RNA polymerase III antibodies and systemic sclerosis: Analysis of the EULAR scleroderma trials and research cohort and possible recommendations for screening. J Rheumatol 2017;44(5):639–47. 28089973 10.3899/jrheum.160817

[25] Steen V. Predictors of end-stage lung disease in systemic sclerosis. Ann Rheum Dis 2003;62(2):97–9. 12525376 10.1136/ard.62.2.97 PMC1754422

[26] Nishimagi E Tochimoto A Kawaguchi Y Satoh T Kuwana M Takagi K Characteristics of patients with early systemic sclerosis and severe gastrointestinal tract involvement. J Rheumatol 2007;34(10):2050–5. 17924608

[27] Dagher JH Scheer U Voit R Grummt I Lonzetti L Raymond Y Autoantibodies to NOR 90/hUBF: Longterm clinical and serological follow-up in a patient with limited systemic sclerosis suggests an antigen-driven immune response. J Rheumatol 2002;29(7):1543–7. 12136917

[28] Liaskos C Marou E Simopoulou T Barmakoudi M Efthymiou G Scheper T Disease-related autoantibody profile in patients with systemic sclerosis. Autoimmunity 2017;50(7):414–21. 28749191 10.1080/08916934.2017.1357699

[29] Mahler M Satoh M Hudson M Baron M Chan JYF Chan EKL Autoantibodies to the Rpp25 component of the Th/To complex are the most common antibodies in patients with systemic sclerosis without antibodies detectable by widely available commercial tests. J Rheumatol 2014;41(7):1334–43. 24931955 10.3899/jrheum.131450

[30] Steen V. Autoantibodies in systemic sclerosis. Semin Arthritis Rheum 2005;35:35–42. 16084222 10.1016/j.semarthrit.2005.03.005

[31] Maddison PJ. Mixed connective tissue disease: Overlap syndromes. Bailliere’s Best Pract Res Clin Rheumatol 2000;14(1):111–24. 10882217 10.1053/berh.1999.0080

[32] D’Aoust J Hudson M Tatibouet S Wick J Mahler M Baron M Clinical and serologic correlates of anti-pm/scl antibodies in systemic sclerosis: A multicenter study of 763 patients. Arthritis Rheumatol 2014;66(6):1608–15. 24577935 10.1002/art.38428

[33] Mimori T. Clinical Significance of Anti-Ku Autoantibodies -A Serologic Marker of Overlap Syndrome ? Intern Med 2002;41(12):1096–8. 12521194 10.2169/internalmedicine.41.1096

[34] Hoa S Hudson M Troyanov Y Proudman S Walker J Stevens W Single-specificity anti-Ku antibodies in an international cohort of 2140 systemic sclerosis subjects: Clinical associations. Medicine (United States) 2016;95(35). 10.1097/MD.0000000000004713 PMC5008592 27583908

[35] Baroni SS Santillo M Bevilacqua F Luchetti M Spadoni T Mancini M Stimulatory Autoantibodies to the PDGF Receptor in Systemic Sclerosis. NEJM 2006;354(25):2667–76. 16790699 10.1056/NEJMoa052955

[36] Berger M Steen VD. Role of anti-receptor autoantibodies in the pathophysiology of scleroderma. Autoimmun Rev 2017;16(10):1029–35. 28778706 10.1016/j.autrev.2017.07.019

[37] Dugar M Cox S Limaye V Gordon TP Roberts-Thomson PJ. Diagnostic utility of anti-Ro52 detection in systemic autoimmunity. Postgrad Med J 2010;86(1012):79–82. 20145055 10.1136/pgmj.2009.089656

[38] Shiboski CH Shiboski SC Seror R Criswell LA Labetoulle M Lietman TM 2016 American College of Rheumatology/European League Against Rheumatism Classification Criteria for Primary Sjögren’s Syndrome: A Consensus and Data-Driven Methodology Involving Three International Patient Cohorts. Arthritis Rheumatol 2017;69(1):35–45. 27785888 10.1002/art.39859 PMC5650478

[39] Bacman S Berra A Sterin-Borda L Borda E. Muscarinic acetylcholine receptor antibodies as a new marker of dry eye Sjögren syndrome. Investig Ophthal Vis Sci 2001;42(2):321–7. 11157861

[40] Giacomelli R Afeltra A Alunno A Baldini C Bartoloni-Bocci E Berardicurti O International consensus: What else can we do to improve diagnosis and therapeutic strategies in patients affected by autoimmune rheumatic diseases (rheumatoid arthritis, spondyloarthritides, systemic sclerosis, systemic lupus erythematosus, antiphospholipid syndrome, and Sjogren’s syndrome)?: The unmet needs and the clinical grey zone in autoimmune disease management. Autoimmun Rev 2017;16(9):911–24. 28705780 10.1016/j.autrev.2017.07.012

